# Does transvaginal natural orifice transluminal endoscopic surgery affect female sexual function?: a prospective cohort study

**DOI:** 10.1186/s12905-023-02566-y

**Published:** 2023-08-02

**Authors:** Dingyu Xu, Li He, Yonghong Lin, Yayu Zhou, Zhaolin Gong, Qian Zhang, Qiannan Hou, Lu Huang

**Affiliations:** Department of Gynecology, Chengdu Women’s and Children’s Central Hospital, School of Medicine, University of Electronic Science and Technology of China, Chengdu, Sichuan China

**Keywords:** Transvaginal Natural Orifice Transluminal endoscopic surgery, vNOTES, Female sexual function

## Abstract

To evaluate the effect of transvaginal natural orifice transluminal endoscopic surgery (vNOTES) on female sexual function. Methods: The trial was registered at the Chinese Clinical Trial Registry (ChiCTR2100050887, 07/09/2021). In this prospective cohort study, we prospectively analyzed the data of the female sexual function index (FSFI) questionnaire of 130 patients who underwent laparoscopy in Chengdu Women’s and Children’s Central Hospital due to gynecological benign diseases. The patients were assigned to the vNOTES group and the control group (underwent traditional laparoscopic surgery or transumbilical laparoendoscopic single-site surgery). Results: There were 4 cases dropout in the vNOTES group and 2 cases dropout in the control group. There was no difference in the ages (31.70 ± 5.02 vs. 30.37 ± 5.74, P>0.05), BMI (body mass index, 21.76 ± 3.16 vs. 23.30 ± 2.69, P>0.05), Education level, surgical types, and FSFI scores (22.31 ± 2.25 vs. 21.55 ± 3.38) between the vNOTES group and the control group before surgery. There was no difference in FSFI scores six months postoperation between the vNOTES group and the control group (21.61 ± 3.22 vs. 20.99 ± 3.26, P>0.05), and there was no difference in FSFI scores pre- and six months postoperation in vNOTES group (21.61 ± 3.22 vs. 22.31 ± 2.25, P>0.05). The time to start sexual life after surgery in the vNOTES group was later than that in the control group (39.34 ± 0.71 d versus 37.86 ± 0.69 d, P < 0.05). Conclusions: vNOTES has no significant adverse effect on female sexual function, however, the time to start sexual life after vNOTES is later than that after trans-abdominal laparoscopy.

## Background

Natural orifice transluminal endoscopic surgery (NOTES) emerges as a significant innovation in the field of minimally invasive surgery during the last decade, which utilizes the natural orifices of the body surface, such as the mouth, anus, vagina, or urethra, to access the peritoneal cavity [1]. This surgical procedure does not leave any incision on the abdominal wall and meets higher aesthetic needs, representing the development trend of minimally invasive technology. In recent years, transvaginal NOTES (vNOTES) has been increasingly applied in many types of gynecologic procedures including adnexal surgery [2], hysterectomy [3], myomectomy [4], sacrocolpopexy [5], and uterosacral ligament suspension [6].

The clinical feasibility of vNOTES in gynecological benign disease surgery have been extensively reported [2–6]. However, relatively little is known about the postoperative complications and the impact on the long-term sexual quality of life of patients. Recent studies [7–10] have shown the impact of vNOTES nephrectomy and cholecystectomy on sexual function, but their conclusions are inconsistent, and the average age of selected patients reaches perimenopausal or postmenopausal age. Hence, the obtained findings are not necessarily suitable for women of childbearing age. Since the vaginal incision is deep into the pelvic cavity in vNOTES, it is still an urgent issue to determine whether the sexual function of patients is affected as a result.

This study is intended to conduct a prospective cohort study on the sexual function of sexually active women undergoing vNOTES for gynecological benign diseases, thereby determining whether vNOTES exerts adverse effects on the sexual function of female patients and further confirming the effectiveness and safety of vNOTES.

## Methods

This study was approved by the Ethics Committee of Chengdu Women’s and Children’s Central Hospital (No: [2020]164). The trial was registered at the Chinese Clinical Trial Registry (ChiCTR2100050887). All participants provided written informed consent after enrollment.

### Inclusion criteria

1. From January 1, 2021 to December 31, 2021, nonporous women of reproductive age undergoing laparoscopic surgery for “benign gynecological diseases” in Chengdu Women’s and Children’s Central Hospital; 2. 18–40 years old; 3. Sexually active women who have fixed sex partners, regular sex.

### Exclusion criteria

1. Patients with a previous history of vaginal or cervical surgery; 2. Patients undergoing hysterectomy or subtotal hysterectomy in this surgery; 3. Patients included in the vNOTES group underwent a conversion from vNOTES surgery to trans-abdominal laparoscopy; 4. malignant tumors suggested by postoperative pathological examination.

### Assignment of the patients

The assignment of the patients is determined by combining the doctor’s assessment and the patient’s wishes. All patients were assigned to the vNOTES group or control group according to different surgical approaches. The control group included traditional laparoscopic surgery and transumbilical laparoendoscopic single-site surgery.

### Questionnaire

The Female Sexual Function Index (FSFI) is a commonly used sexual functioning questionnaire developed by Rosen et al. [11] in 2000. In 2011, Sun et al. [12] translated it into Chinese and verified its satisfactory reliability and effectiveness for the Chinese population. In this study, FSFI was used to assess female sexual function, including six dimensions of sexual desire, sexual arousal, vaginal lubrication, orgasm, sexual satisfaction, and sexual pain, with a total score of 36 points.

**Table 1:** Baseline characteristics

	vNOTES group (n = 65)	Control group (n = 65)	p
Age	31.70 ± 5.02	30.37 ± 5.74	0.413
BMI ^a^	21.76 ± 3.16	23.30 ± 2.69	0.077
Education level [n(%)]	Bachelor degree or above 43(66.15)	Bachelor degree or above 34(52.31)	0.076
	Below bachelor degree 22(33.85)	Below bachelor degree 31(47.69)	
surgical type [n(%)]	Ovarian cystectomy 19(29.23)	Ovarian cystectomy 25(38.46)	0.122
	Fallopian tube-related surgery ^b^ 39(60.00)	Fallopian tube-related surgery 27(41.54)	
	Uterine related surgery ^c^ 7(10.77)	Uterine related surgery 13(20.00)	
FSFI score pre-operation	22.31 ± 2.25	21.55 ± 3.38	0.143

a: BMI: body mass index, vNOTES: transvaginal natural orifice transluminal endoscopic surgery, FSFI: female sexual function index

b: Fallopian tube-related surgery including: salpingectomy, tubal pregnancy fenestration and embryo extraction, tubal ligation, and salpingostomy

c: Uterine related surgery: myomectomy and excision of adenomyoma of uterus

### Sample size

The two-sided test was adopted, setting the level of significance alpha at 5% (α = 0.05) and the power of the sample at 80% (β = 0.20). According to the pre-test results, the FSFI scores of the exposure group and control group were 20.87 ± 6.87 and 23.75 ± 5.50 respectively. Considering a dropout rate of 10%, the estimated sample size was N1 = N2 = 65 cases by using PASS 15 software.

**Table 2:** FSFI score of patients in vNOTES group pre- and post-operation

	Preoperation	3 months postoperation	t	*p*	6 months postoperation	t	*p*
Sexual desire	3.41 ± 1.12	3.35 ± 1.22	0.298	0.767	3.13 ± 1.18	-1.576	0.120
Sexual arousal	3.45 ± 1.00	3.33 ± 0.77	0.761	0.450	3.42 ± 0.74	-0.276	0.784
Vaginal lubrication	3.87 ± 0.78	3.82 ± 0.86	0.341	0.735	3.91 ± 0.89	0.367	0.715
Orgasm	3.95 ± 0.90	3.86 ± 1.01	0.455	0.651	3.70 ± 0.95	-1.624	0.110
Sexual satisfaction	3.82 ± 1.05	3.91 ± 0.93	-0.499	0.620	3.84 ± 1.05	0.097	0.923
Sexual pain	3.80 ± 0.93	3.77 ± 1.03	0.170	0.866	3.61 ± 0.86	-1.497	0.140
Total	22.31 ± 2.25	22.06 ± 3.07	0.512	0.610	21.61 ± 3.22	-1.751	0.085

The primary outcome was the FSFI score at 3 and 6 months after surgery. The secondary outcome was the time to start sexual life after surgery. The patient’s age, height, weight, surgical type, the time to start sexual life after surgery, the preoperative FSFI score, and the postoperative FSFI score after 3 and 6 months were collected.

### Statistical analysis

SPSS 20.0 statistical software was used for statistical analysis. The measurement data were expressed as mean ± standard deviation such as age, body mass index and FSFI score. The data conforming to normal distribution and homogeneity of variance adopted the *t*-test for comparisons between two groups. The measurement data of non-normal distribution were described by the median, and the Wilcoxon rank sum test was used for comparisons between two groups. The counting data were described by frequency (percentage), and the chi-square test was used for comparisons between two groups. The preoperative and postoperative FSFI scores of the same patients were compared by paired *t*-test. All statistical analyses in this study were conducted by two-sided tests, and P < 0.05 was indicative of statistical significance.

## Results

**Fig. 1:**
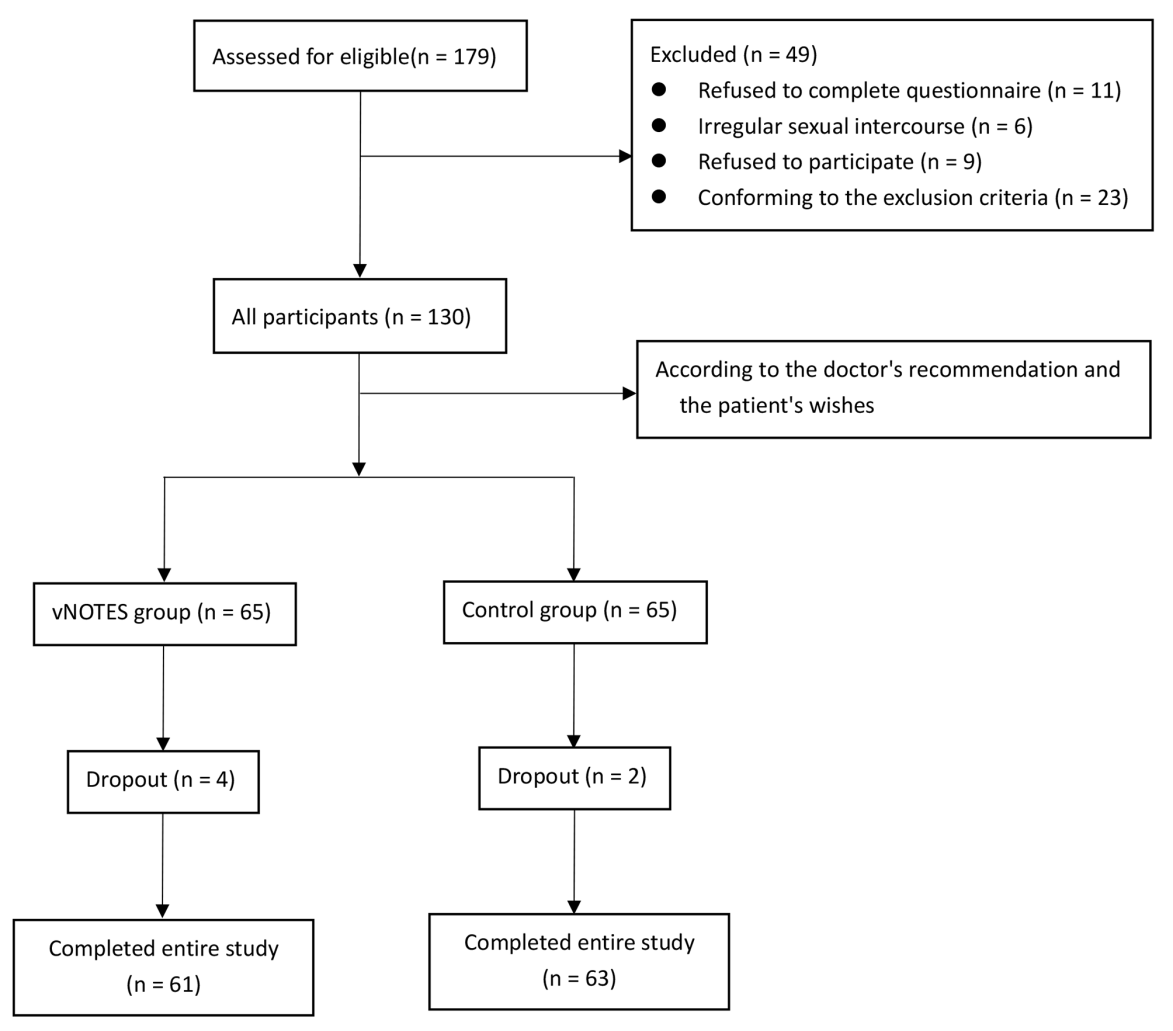
Subject screening and completion flowchart

A total of 130 patients were included in this study including 65 in the vNOTES group and 65 in the control group. Among them, 3 patients in the vNOTES group and 2 patients in the control group dropped out during the 3 months of follow-up due to incorrect phone numbers. Moreover, 1 patient in the vNOTES group was dropped out during the 6 months of follow-up, because she did not have sexual activity in the last month and refused to complete the questionnaire. Finally, 61 patients in the vNOTES group and 63 patients in the control group were followed up (Fig. 1).

**Table 3:** FSFI score of patients in control group pre- and post-operation

	Preoperation	3 months postoperation	t	*p*	6 months postoperation	t	*p*
Sexual desire	3.24 ± 1.16	3.13 ± 1.13	0.495	0.622	3.30 ± 1.26	0.403	0.688
Sexual arousal	3.29 ± 0.72	3.32 ± 0.84	-0.225	0.823	3.26 ± 0.58	-0.374	0.709
Vaginal lubrication	3.69 ± 0.87	3.71 ± 0.93	-0.122	0.903	3.66 ± 0.87	-0.301	0.764
Orgasm	3.82 ± 1.08	3.59 ± 1.07	1.182	0.242	3.60 ± 0.74	-1.389	0.170
Sexual satisfaction	3.74 ± 0.98	3.64 ± 1.06	0.504	0.616	3.61 ± 0.76	-0.985	0.328
Sexual pain	3.78 ± 0.98	3.61 ± 1.00	0.910	0.366	3.56 ± 0.84	-1.550	0.126
Total	21.55 ± 3.38	21.00 ± 2.95	0.958	0.342	20.99 ± 3.26	-1.217	0.228

The average age of included patients was 31.70 ± 5.02 and 30.37 ± 5.74 years old in the vNOTES group and control group respectively, and there was no statistically significant difference between the two groups (P = 0.413). The average body mass index (BMI) of patients in the vNOTES group and control group was 21.76 ± 3.16 Kg/m2 and 23.30 ± 2.69 Kg/m2 respectively, with no significant difference between the two groups (P = 0.077). The preoperative FSFI score of patients in the vNOTES group and control group was 22.31 ± 2.25 and 21.55 ± 3.38 respectively, with no statistically significant difference (P = 0.143). There was no statistically significant difference between the two groups in terms of education level and surgical type (Table 1).

The total FSFI score of patients in the vNOTES group before surgery, 3 months after surgery, and 6 months after surgery was 22.31 ± 2.25, 22.06 ± 3.07, and 21.61 ± 3.22 respectively. There was no statistically significant difference in the total score of FSFI at 3 months and 6 months after surgery compared with the total score of FSFI before surgery (P = 0.085) (Table 2).

The total FSFI score of patients in the control group before surgery, 3 months after surgery, and 6 months after surgery was 21.55 ± 3.38, 21.00 ± 2.95, and 20.99 ± 3.26 respectively. There was no statistically significant difference in the total score of FSFI at 3 months and 6 months after surgery compared with the total score of FSFI before surgery (P = 0.228) (Table 3).

Compared with that in the control group, the total score of FSFI in the vNOTES group had no statistical difference at 3 months and 6 months after surgery. The time to start sexual life after surgery in the vNOTES group and control group was 39.34 ± 0.71 days and 37.86 ± 0.69 days respectively, with a significant difference (P = 0.001) (Table 4 ).

**Table 4:** Comparison between vNOTES group and control group postoperation

	vNOTES group	Control group	p
FSFI scores 3 months postoperation	22.06 ± 3.07	21.00 ± 2.95	0.054
FSFI scores 6 months postoperation	21.61 ± 3.22	20.99 ± 3.26	0.288
Time to start sexual life postoperation (day)	39.34 ± 0.71	37.86 ± 0.69	0.001

## Discussion

This study was designed to assess the effect of vNOTES on female sexual function. Our results revealed no significant difference in sexual function before and after surgery in patients undergoing vNOTES, and also no significant difference in sexual function after surgery between patients receiving vNOTES and transabdominal laparoscopy. However, the time of starting sexual life after vNOTES is later than that after transabdominal laparoscopy.

vNOTES is a novel surgical approach emerging in recent years. Gettman MT et al. [13] first performed vNOTES nephrectomy in a porcine model in 2002. Marescaux et al. [14] reported vNOTES cholecystectomy and applied vNOTES technology to the human body for the first time in 2007. In the following 10 years, vNOTES has flourished in general surgery and urology, which attains satisfactory effects in nephrectomy [15], prostatectomy [16], appendectomy [17], sigmoid colon cancer surgery [18], etc. However, the application of vNOTES in gynecological surgery is slightly delayed. Until 2012, Lee CL [19] and Ahn KH [20] reported the application of vNOTES for adnexectomy. Since then, vNOTES has been widely performed in gynecology. So far, vNOTES has been used in various gynecological surgeries, including transgender surgery[2–6, 21–23]. vNOTES combines laparoscopic surgery with conventional vaginal surgery. Transvaginal surgery is a special type of minimally invasive surgery with a long history, but surgeons performing this procedure are challenged by restrictions on the view field and operative scope. The incorporation of vNOTES enables the observation of the entire pelvic and abdominal cavity, thus significantly expanding the scope of access of transvaginal surgery [24]. Compared with traditional laparoscopy, vNOTES has other advantages in addition to cosmetic effects. For example, the vaginal incision is more ductile than the abdominal incision, making the removal of solid tumors, such as teratoma or myoma, faster and easier. In addition, vNOTES leads to less postoperative pain than traditional laparoscopy [25], which may be because the vaginal fornix is innervated by visceral nerves, so patients feel less pain after surgery compared to traditional skin incisions. In our previous research [26], 1147 patients who accepted vNOTES were included. A total of 38 patients had complications, and the total complication rate was 3.31%. There were 27 cases of grade I, 4 cases of grade II and 7 cases of grade III complications and without grade IV, V complications according to Clavien-Dindo classification. And 18 patients were converted to conventional or transumbilical single-site laparoscopic surgery. The conversion rate was 1.57%.

The current studies relevant to vNOTES have reported its feasibility and effectiveness, but its long-term safety and impact on the quality of life of patients have not been deeply discussed. This study used the validated FSFI questionnaire to evaluate six dimensions of sexual function (sexual desire, arousal, vaginal lubrication, orgasm, satisfaction, and sexual pain) and found that there was no significant difference in sexual function before and after surgery in the vNOTES group. This may be related to the anatomical structure and nerve distribution of the female lower genital tract. The vaginal innervation is concentrated to the far and front along the vaginal wall, leaving sparse sensory innervation of the posterior fornix, while the incision of vNOTES is just located at the posterior fornix of the vagina, so it has little impact on it [27, 28]. Gynecologists have attempted to explore the impact of transvaginal surgery on sexual function for many years. Some scholars declare that [29, 30] traditional vaginal hysterectomy does not affect female sexual function. A self-control study on pre- and post- vNOTES cholecystectomy also shows that vNOTES has no effect on female sexual function [7]. On the contrary, Sener et al. [10] believe that sexual function can decline after vNOTES nephrectomy and suggest that postoperative nursing should include some methods for restoring sexual function. However, the average age of participants included in the above study is 52.72 years old, which may lead to inconsistent results in sexually active women.

There is currently no randomized controlled study on the optimal time to start sexual life after vNOTES. According to previous studies, the suggested time of starting sexual life is 2 weeks after surgery [31–34]. However, some studies also indicate that it takes longer to start sexual life after vNOTES. One study from Switzerland suggests that the time to start sexual life after vNOTES cholecystectomy should be 3–6 weeks [35], and another study from Turkey suggests that the time to start sexual life after vNOTES nephrectomy should be 6 weeks [10]. Actually, the time for each patient to start sexual life after surgery is different. A prospective cohort study by Yassa M et al. [27] shows that 75% of patients undergoing vNOTES bilateral salpingectomy have sexual activity during the one-month period of follow-up, with no complications caused by sexual activity. A Japanese study [36] performed a retrospective analysis of transvaginal ovarian cystectomy (conventional vaginal surgery or vNOTES) and found that the median time for patients to start sexual life after surgery was 2 months, ranging from 1 month to 12 months. In this study, the time to start sexual life in the vNOTES group was 39.34 ± 0.71 days, which was significantly later than that in the control group. In fact, patients are suggested to start intercourse 1 month after both transabdominal laparoscopic surgery and vNOTES in our hospital. Therefore, the late time to start sexual activity after vNOTES surgery may be due to patients’ concerns that intercourse may hinder the healing of vaginal incisions. The optimal time to start sexual life after vNOTES needs to be confirmed by further randomized controlled studies.

However, this study has a limitation. We only assessed changes in women’s sexual function after surgery. The sexual life is mutual, and male sexual satisfaction should also be valued. For example, the Arizona sexual experiences scale (ASEX) can be used to assess the sexual function of both partners. Therefore, in the future more large sample randomized controlled studies are needed to further evaluate the impact of vNOTES on the sexual satisfaction of both sexual partner.

## Conclusions

vNOTES has no adverse impact on female sexual function postoperatively, while it extends the time to start sexual life compared to trans-abdominal laparoscopy. The optimal time to start sexual life after surgery needs to be determined by further large-sample randomized controlled studies.

## Data Availability

The data that support the findings of this study are available on request from the corresponding author. The data are not publicly available due to privacy restricions.
